# Parkinson’s disease and depression

**DOI:** 10.1192/j.eurpsy.2021.643

**Published:** 2021-08-13

**Authors:** A. Maamri, H. Ghabi, H. Zalila

**Affiliations:** 1 Faculty Of Medincine Of Tunis, RAZI HOSPITAL, TUNIS, Tunisia; 2 Psychiatry, Outpatient Service, Razi Hospital, Manouba, Tunisia; 3 Outpatients Ward Of Psychiatry, Razi hospital, Manouba, Tunisia

**Keywords:** parkinson’s disease, Depression

## Abstract

**Introduction:**

Parkinson’s disease has long been considered as a neurodegenerative disorder of pure motor expression. Motor dysfunction in Parkinson’s disease and other parkinsonian disorders is frequently accompanied by nonmotor signs and symptoms, including cognitive impairment, apathy, anxiety, and depression. Among psychiatric disorders comorbid with Parkinson’s disease, depression is probably the most important in terms of frequency and impact.

**Objectives:**

The aim of this presentation was to illustrate the importance of considering depressive symptoms in patients with Parkinson’s disease.

**Methods:**

A case report describing a patient with depressive symptoms in a patient with Parkinson’s disease and literature review.

**Results:**

We report a case of a 57-year-old woman who presented symptoms of Parkinson’s disease for two years. She was treated with Benserazide (Madopar). She was referred to our department for depressive symptomatology. The patient suffered from fatigue, insomnia, loss of sexual desire, sadness, anhedonia, and social withdrawal during the last three months. The diagnosis of depression was not immediately retained. Finally, a major depressive episode was diagnosed. Fluoxetine (20mg per day) was prescribed with clinical improvement.

**Conclusions:**

The diagnosis of a depressive episode is most often complex, due to an overlap symptomatic of both disorders. The depression comorbid to Parkinson’s disease because of its frequency and impact, requires specific identification and management early.

